# Hepatoprotective Effects of Aqueous Extract of *Opilia celtidifolia* (Opiliaceae) Leaves against Ethanol-Induced Liver Damage in Rats

**DOI:** 10.1155/2020/6297475

**Published:** 2020-10-24

**Authors:** A. P. Amang, E. Kodji, C. Mezui, M. P. Baane, G. T. Siwe, T. M. Kuissu, J. Emakoua, P. V. Tan

**Affiliations:** ^1^Department of Biological Sciences, Faculty of Science, University of Maroua, P.O. Box 814, Maroua, Cameroon; ^2^Department of Biological Sciences, Higher Teachers' Training College, University of Yaoundé I, P.O. Box 47, Yaoundé, Cameroon; ^3^Socio-Medical Center of National Social Insurance Fund, P.O. Box 120, Maroua, Cameroon; ^4^Department of Animal Biology and Physiology, Faculty of Science, University of Yaoundé I, P.O. Box 812, Yaoundé, Cameroon; ^5^Institute of Medical Research and Study of Medicinal Plants, P.O. Box 13033, Yaoundé, Cameroon

## Abstract

**Objective:**

*Opilia celtidifolia* is a plant used in Cameroonian ethnomedicine to cure jaundice. The aim of this study was to evaluate the hepatoprotective activity of *O. celtidifolia* leaves aqueous extract in rats subjected to ethanol-induced liver damage. *Material and Methods*. 36 rats including 18 males and 18 females were divided into 6 groups of 3 males and 3 females each, namely, 3 control groups (normal, negative, positive) and 3 test groups. The normal and negative control groups were given distilled water (5 ml/kg), the positive control group received silymarin (100 mg/kg), and the test groups were given *O. celtidifolia* extract at the doses of 100, 200, and 400 mg/kg. All groups, except the normal control, received concomitantly and daily 40% ethanol (4 g/kg) for 3 weeks to induce hepatotoxicity. Biochemical parameters such as alanine aminotransferase (ALT), aspartate aminotransferase (AST), alkaline phosphatase (ALP), bilirubin, and lipid profile (total cholesterol, HDL, LDL, and triglycerides) were evaluated. Histological sections of the liver, kidneys, and lungs were examined. Qualitative and quantitative phytochemical analysis of the extract were carried out.

**Results:**

The groups treated with the extract at the doses of 200 and 400 mg/kg showed a significant decrease (*p* < 0.001) of transaminases (ALT and AST), ALP, and bilirubin compared with the negative control. These results were confirmed by observation of histological sections of the liver that confirmed protective action of extract against ethanol-induced hepatocellular injury.

**Conclusion:**

*O. celtidifolia* possesses hepatoprotective properties that could be related to its high content of tannins and saponins in the leaves aqueous extract.

## 1. Introduction

The liver is one of the animal body organs that ensures the detoxification and elimination of substances found in the blood [1]. When blood levels of alcohol exceed the detoxifying capacity of the liver, liver cells are attacked causing hepatitis (inflammation of liver cells). Liver damage can also be caused by viruses or toxic substances from bioactivation of metabolites of drugs [2].

According to the World Health Organization [3] (WHO), in 2012, 325 million people around the world were suffering from viral hepatitis, with 95% of them being unaware of their infection, and 1.4 million hepatitis-related deaths occur each year. Hepatitis is the 10^th^ leading cause of death in the world, with Africa being the continent with the highest prevalence (6.15%). The prevalence in Cameroon is 11%, ranking the latter among the most affected countries in Africa after Egypt (18%) and Rwanda (14%) [3]. According to the Ministry of Public Health statistics in 2016, 200,000 people were affected by viral hepatitis in Cameroon. It is also established that toxic substances cause about 10% of acute liver failures [4]. In the case of alcoholic hepatitis, 13.5% of people worldwide who regularly drink alcohol are suffering from hepatitis. Consumption of alcohol above 25 g/day increases the cirrhosis risk by 1.5 to 3.6% [5]. The complications of alcoholic hepatitis depend on the frequency and duration of consumption as well as the dose of alcohol ingested [6].

The treatment of viral hepatitis by modern medicine consists of taking interferons: Lamivudine, Tenofovir, Adefovir, and Ribavirin [7]. Nonviral hepatitis is treated by the suppression of the cause, accompanied by the taking of hepatoprotective agents such as Simepar or Silybon [8]. Due to inaccessibility of treatment and the numerous drug side effects, people in developing countries are increasingly embracing the use of medicinal plants to treat these pathologies.

*Opilia celtidifolia* belongs to the Opiliaceae family. It is found in Mali, Senegal, Nigeria, and the Far North Region of Cameroon [9]. The phytochemical screening of the leaves aqueous extract shows phlobatannins, alkaloids, terpenoids, and anthraquinones [10]. It is one of the plants commonly used by the population of the Far North Region of Cameroon for its many therapeutic virtues including the treatment of jaundice, chest, and abdominal pains [9]. Ethnobotanical studies show that the leaves of *O. celtidifolia* are used for the traditional treatment of hepatitis in Benin [11] and jaundice in Mali [12], and according to the work of Traoré et al., the decoction leaves of *Opilia celtidifolia* are taken to treat hepatitis [13]. Hence, the objective of this study was to evaluate the hepatoprotective activity of *O. celtidifolia* leaves aqueous extract in rats.

## 2. Materials and Methods

### 2.1. Plant Material

*Opilia celtidifolia* leaves were harvested in July 2018, around Mokolo, Mayo-Tsanaga Division, Far North Region of Cameroon (10°44′32.78″N 13°48′8.17″E). The sample was then authenticated at the Herbarium of the Garoua Wildlife School by Mr. Vounserbo through comparison with the existing voucher specimen recorded under N^o^ HEFG/1962. These leaves were dried in the shade at room temperature, then ground in a clean mortar, and sieved. The fine powder obtained was used for the preparation of the aqueous extract.

### 2.2. Animal Material

Thirty-six albino Wistar rats of both sexes, 7 to 8 weeks old, with an average body weight of 100 ± 20 g were obtained from the Animal House of the Laboratory of Biological Sciences of the University of Ngaoundere. These animals had not been subjected to any previous experimentation. They were acclimatized in the Laboratory of Animal Physiology and Pharmacology of the University of Maroua for two weeks. The rats were subjected to natural day/night cycle at room temperature and fed with a standard diet, with free access to tap water. Prior authorization for the use of laboratory animals in this study was obtained from the Cameroon National Ethics Committee (Registration number FWA-IRB00001954). The use, handling, and care of animals were done with adherence to the European Convention for the Protection of Vertebrate Animals used for experimental and other purposes (ETS-123), with particular attention to Part III, articles 7, 8, and 9 [19].

### 2.3. Preparation of *O. celtidifolia* Leaf Aqueous Extract

The decoction of *O. celtidifolia* was prepared by boiling 300 g of *O. celtidifolia* powder in 3 liters of distilled water for 15 minutes. The mixture was allowed to cool and filtered using Whatman filter paper №3. The filtrate was evaporated in a ventilated oven at 50°C for 24 hours. The dry extract obtained weighed 25.9 g, representing an extraction yield of 8.63%. This extract was stored at 4°C in a refrigerator throughout the experiment [15].

### 2.4. Qualitative and Quantitative Phytochemical Tests

The protocol described by Harborne [16] was used for qualitative phytochemical tests of the aqueous extract of *O. celtidifolia* leaves in order to detect the presence or absence of secondary metabolites classes such as flavonoids, phenols, tannins, proteins, saponins, and coumarins.

The saponins and tannins contents were determined using the method described by Brunner [17, 18]. The contents of saponins and tannins were determined from the linear regression equation curve plotted with the standard solutions, galactose and catechin, respectively.

### 2.5. Animal Distribution and Treatment

After a two-week acclimatization period, the 36 rats were randomized and divided into 6 groups of 6 rats including three males and three females. Males and females of the same group were kept separately in cages as follows: group I: normal control, which received distilled water (5 ml/kg/day) for 21 days; group II: negative control treated with ethanol (4 g/kg/day) given concomitantly with distilled water (5 ml/kg/day) for 21 days; group III: positive control treated with ethanol (4 g/kg/day) and concomitantly treated daily with Simepar (oral route) at the dose of 100 mg/kg for 21 days; groups IV, V, and VI: treated, respectively, with the extract of *O. celtidifolia* at the doses of 100, 200, and 400 mg/kg/day given concomitantly with 40% ethanol (4 g/kg/day) for 21 days. The ethanol was used to induce hepatotoxicity [19].

### 2.6. Animal Euthanasia and Biochemical Assays

The next day following the end of the treatment period, all the rats were sacrificed by decapitation, and the blood was collected in dry tubes and centrifuged at 4000 rpm for 15 minutes at 4°C. The serum obtained was stored at −20°C for the measurement of biochemical parameters. The transaminases (ALT and AST) were assayed by the method described by Reitman and Frankel [20] using Giesse Diagnostics AST/GOTSL Kits and Atlas Medical GOT Kit. Serum alkaline phosphatase (ALP) concentration was determined according to the method described by Otto et al. [21] using Q-SLAP Alkaline Phosphatase (DEA) Kit, Biotec Diagnostics Ltd., UK.

Enzyme linked immunosorbent assay kits were used to determine enzyme profiles. Bilirubin was determined by the colorimetric method described by Doumas et al. [22] using Biotec Diagnostics Ltd. Kit, UK, and Direct DMSO Kits. Total cholesterol was determined according to the method described by Allain et al. [23] using Hospitex Diagnostics SARL cholesterol kits. HDL cholesterol levels were determined according to the method of Lopes-Virella et al. [24] using Q-SLAP HDL Cholesterol Kit, Biotec Diagnostics Ltd., UK. The concentration of LDL cholesterol in the samples was determined by the equation of Fridewald et al. [25]. Triglyceride concentrations were determined according to the method described by Bucolo and David [26] using Giesse Diagnostics SL, GPO-POD Kits. The colorimetric method described by Gornall et al. [27] was used to assay total protein.

### 2.7. Histopathological Analysis

Liver, kidney, and lung tissue samples were collected, weighed, rinsed in saline (0.9% NaCl), and stored in 10% buffered formaldehyde solution for histopathological examination according to the methods described by Rhiouani et al. [28] and Lienou et al. [29].

### 2.8. Statistical Analysis

The data were analyzed using Analysis of Variances (ANOVA), followed by the Bonferroni comparison test as a posttest using GraphPad Prism software, version 5.03, and the results were expressed as mean ± standard error on mean (SEM).

## 3. Results

### 3.1. Phytochemical Analysis of *O. celtidifolia*

Qualitative phytochemical screening of the aqueous extract of *O. celtidifolia* leaves revealed the presence of some classes of bioactive compounds such as tannins and saponins (Table 1).

The quantification of bioactive compound revealed that 100 g of dry extract contains 6.48 Eqg Galac/100 g of saponin and 9.41 Eqg Catech/100 g of tannin (Table 2).

### 3.2. Effects of *O. celtidifolia* on Body Weight

Table 3 shows the changes in body weight (g/animal/week) of rats. There was no significant difference in body weight of rats treated with *O. celtidifolia* extract compared to the negative control.

### 3.3. Effects of *O. celtidifolia* on Some Serum Parameters of Liver Function

Table 4 shows that the daily administration of ethanol (4 g/kg) for 21 days induced a significant increase of transaminases levels (ALT and AST), alkaline phosphatase, and bilirubin of the negative control group compared to the normal controls. Treatment with *O. celtidifolia* extract resulted in a decrease in serum levels of these parameters in the different groups. However, the decrease in ALT was significant (*p* < 0.001) only at the doses of 100 and 200 mg/kg. As for AST at 400 mg/kg, the extract showed slight tendency to raise transaminase levels compared to the dose of 200 mg/kg (ALT and AST). The extract induced a significant decrease (*p* < 0.001) in ALP and bilirubin only at the dose of 100 mg/kg in rats compared to the negative controls.

### 3.4. Effects of *O. celtidifolia* on the Lipid Profile and Total Protein

Table 5 shows the results of the lipid profiles and total protein levels of the different groups. There was a significant decrease (*p* < 0.001) of total cholesterol in groups treated with extract at the dose of 400 mg/kg. A significant decrease in total protein was observed in the negative control group (*p* < 0.01) compared to the normal control. However, protein values remained close to the normal control levels in the groups treated with extract.

### 3.5. Effects of *O. celtidifolia* on Histology

**Figure 1:**
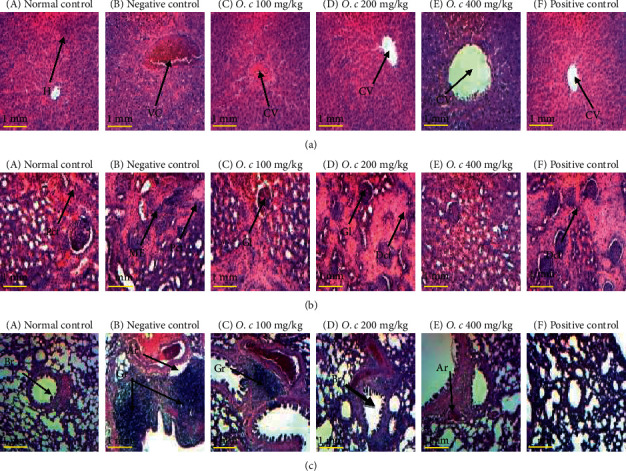
Histology of liver (a), kidneys (b), and lungs (c) of male rats (hex 100). O. c: *O. celtidifolia*, CV: centrilobular vein, VC: vascular congestion, PV: portal vein, H: hepatocyte, LI: leukocyte infiltration, Dct: distal convoluted tubule, Pct: proximal convoluted tubule, Gl: glomerulus, ME: mesangial expansion, Br: bronchiole, Gr: granuloma, Ar: arteriole.

Microphotographs of the liver of normal male and female rats showed normal hepatic parenchyma architecture with hepatocytes arranged in cobblestones around the centrilobular vein (Figures 1(a)A and 2(a)A). Negative control groups showed liver alterations characterized by hepatic cytolysis in rats (Figure 1(a)B and 2(a)B). Rats treated with the extract at the doses of 200 (Figures 1(a)D and 2(a)D) and 400 mg/kg (Figures 1(a)E and 2(a)E) and Simepar (Figures 1(a)F and 2(a)F) showed an improvement in liver structures similar to the normal controls rats.

Histology of renal tissues in normal rats showed a normal architecture of glomeruli, urinary spaces, and proximal and distal convoluted tubules (Figures 1(b)A and 2(b)A). Negative control groups showed renal cell changes characterized by mesangial expansions (Figures 1(b)B and 2(b)B) compared to normal controls. Rats treated with the extract at the doses of 100 (Figures 1(b)C and 2(b)C) and 400 mg/kg (Figures 1(b)E and 2(b)E) and Simepar (Figures 1(b)F and 2(b)F) showed an improvement in kidney architecture structures approximating that of normal control in both sexes.

Lung microphotographs of normal rats showed normal architecture of bronchioles, arterioles, and alveolar sacs (Figures 1(c)A and 2(c)A). Negative control group (Figures 1(c)B and 1(c)B) showed inflammatory granulomas compared to normal control. These alterations were improved in groups treated with the *O. celtidifolia* leaf aqueous extract in males, particularly at the doses of 200 and 400 mg/kg in females (Figures 1(c)D and 1(c)E, respectively). The rats treated with Simepar (Figures 1(c)F and 2(c)F) showed an improvement in lung histology similar to normal controls.

## 4. Discussion

The liver is subject to xenobiotic-induced injury because of its central role in the metabolism of xenobiotics, its portal location in the circulation, and its anatomical and physiological structure. Daily administration of ethanol (40%) at a dose of 4 g/kg for 21 days to rats induced a significant increase (*p* < 0.001) of ALT and AST in the negative control groups compared to normal control rats. Increased serum levels of ALT and AST are clinical signs of hepatic injury resulting from loss of hepatocyte membrane integrity, with release of these enzymes into the serum [30]. Thus, serum levels of ALT and AST activity can be used to indirectly evaluate the integrity of liver tissue after exposure to certain toxic agents [31]. Therefore, AST appears in higher concentrations in a number of tissues (liver, kidneys, heart, and pancreas) and is released slowly in comparison to ALT. However, since ALT is localized primarily in the cytosol of hepatocytes, this enzyme is considered a more sensitive marker of liver inflammation or damage than AST and within limits can provide a quantitative assessment of the degree of damage sustained by the liver [32]. The significant decrease (*p* < 0.01) of ALT and AST levels observed in rats treated with the extract at 200 and 400 mg/kg, compared to the negative control, suggests inhibition of hepatocellular injury by the extract. These results are similar to those of Ngueguim et al. [33], who showed that daily administration of the aqueous extract of *Dichrocephala integrifolia* to rats followed by ingestion of ethanol for 14 days induced a significant decrease in transaminases. It was suggested that the extract either protected cell membranes or counteracted the deleterious effects of ethanol via cytochrome P450 inhibition or stimulation of the gene responsible for hepatocyte regeneration; this is because of the presence of classes of bioactive compounds in the extract such as tannins and saponins revealed by qualitative and quantitative phytochemical analysis.

In cases of hepatic toxicity, serum bilirubin levels rise due to insufficient absorption of unconjugated bilirubin by the liver. This explains the high bilirubin levels in the negative controls rats compared to the normal controls. This biliary constituent reaches the plasma through tight junctions between the biliary canaliculi and sinusoids [34]. The groups treated with *O. celtidifolia* extract showed a significant (*p* < 0.01) decrease of bilirubin levels at the doses of 100, 200, and 400 mg/kg. These results are similar to those of Dahiru and Obidoa [35], who demonstrated that daily administration of Z*iziphus mauritiana* bark aqueous extract for 6 weeks resulted in bilirubin decrease following induction of hepatocellular carcinoma with ethanol. This decrease may be linked to the presence of saponins in the extract capable of protecting the membranes of hepatocytes [36]. The significant decrease (*p* < 0.001) of alkaline phosphatase (ALP) observed in the rats treated at 400 mg/kg could be a clinical indication of the inhibition of cholestasis by the extract. These results corroborate those obtained by Dahiru and Obidoa [35] who demonstrated that daily administration of Z*iziphus mauritiana* bark aqueous extract for 6 weeks resulted in a decrease of ALP and bilirubin in the serum of the rats following induction of hepatotoxicity with ethanol. They suggested that the decline in these parameters could be due to the presence of phytoconstituents such as tannins capable of counteracting the toxic effects of alcohol on the hepatocyte membranes.

Ethanol consumption increases lipogenesis, leading to excessive synthesis of fatty acids and triglycerides, which contributes to the accumulation of fat in the liver [36]. It also inhibits the activity of lipases involved in the transformation of lipoproteins and triglycerides [37]. Inhibition of these enzymes induces changes in the lipid profile (steatosis). Evaluation of these parameters provides information on the adverse effects of ethanol on lipoprotein and triglyceride transformation enzymes [33].

In this study, only the dose of 400 mg/kg of the extract showed a significant decrease of total cholesterol and triglyceride in rats compared to the negative control. HDL and LDL cholesterol remained close to normal values although extract doses of 100–200 mg/kg tended to reduce their values. Proinflammatory cytokines, particularly TNF-*α*, are involved in Kupffer cell activation, hepatic necrosis, endothelial damage, and tissue recruitment of neutrophils in alcoholic liver disease [36]. These results are in line with those obtained by Ngueguim et al. [33], who demonstrated that daily administration of *Dichrocephala integrifolia* leaves aqueous extract to rats followed by ethanol intoxication for 14 days lowered cholesterol and triglyceride levels. They suggested that these effects would be due to the presence of classes of bioactive compounds such as tannins in the extract capable of stabilizing and protecting the integrity of the hepatocyte membrane.

Several proteins synthesized in the liver are found in the blood [38]. Administration of extract restored the serum total protein levels around the normal values after the decrease observed in negative controls. These results could be due to the presence of compounds such as tannins that are able to stimulate genes responsible for protein biosynthesis.

Macroscopic observation of the main organs revealed no abnormalities. Histopathological studies of the liver showed that the architecture of the liver was intact in normal controls; hepatocytes, sinusoids, central veins, and portal veins occur within the normal range in both sexes. Negative control groups showed partial loss of hepatic architecture. The centrilobular vein appeared to be slightly dilated, and the hepatocytes showed degeneration between the lobules. Sinusoids were within normal limits, and there was moderate lymphocyte infiltration in response to inflammation in the periportal region, with overflows in adjacent hepatocytes associated with hemorrhage. These different manifestations are due to the deleterious effects of ethanol, which, during its metabolism, releases acetaldehyde which is potentially toxic to the membrane of hepatocytes [39]. In the group treated with silymarin, the architecture of the liver was preserved; several hepatocytes showed signs of regeneration and slight lymphocyte infiltration. This confirms the hepatoprotective effects of silymarin against the deleterious effects of ethanol. The group treated with the aqueous leaf extract of *O. celtidifolia* at the dose of 200 mg/kg showed no lymphocyte infiltration in male and female rats with a liver architecture close to normal. This suggests that *O. celtidifolia* at this dose could significantly protect hepatocytes from the toxic effects of ethanol. These effects are illustrated by increased hepatocyte regeneration and moderate degeneration, which might be related to the fact that the extract contains bioactive compounds whose hepatoprotective effects are well known.

The histological study of lung and kidney tissues in male and female rats revealed that the negative control groups showed signs of lung and renal cell damage characterized by the formation of inflammatory granulomas and mesangial expansions in both sexes compared to normal controls. These effects are thought to be due to the deleterious effects of excessive ethanol on lung and kidney tissues. The groups treated with extract at the doses of 200 and 400 mg/kg as well as animals treated with silymarin showed an improvement in lung and renal structures close to those of normal controls, suggesting protection of these tissues against the deleterious effects of ethanol by the plant extract.

## 5. Conclusions

This study shows that *O. celtidifolia* extract decreased serum levels of (ALT and AST) transaminases, alkaline phosphatase, bilirubin, triglyceride, and total cholesterol. The extract also prevented histopathological changes in the liver, kidneys, and lungs due to the toxicity of ethanol. The hepatoprotective effects observed could be due to the presence of tannins and saponins.

## Figures and Tables

**Figure 2:**
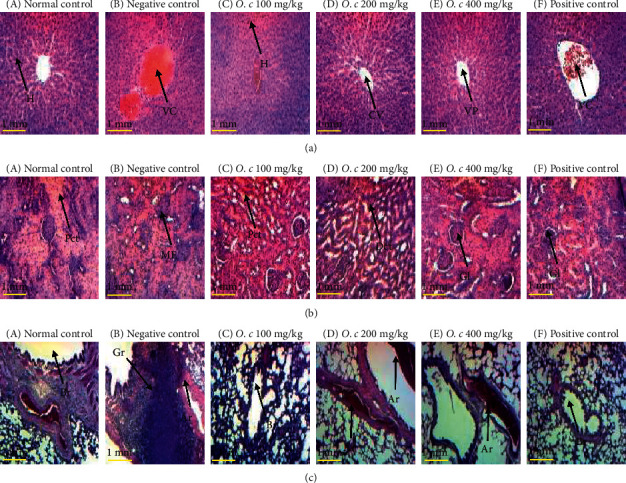
Histology of liver tissue (a), kidneys (b), and lungs (c) of female rats (hex 100). O. c: *O. celtidifolia,* CV: centrilobular vein, VC: vascular congestion, PV: portal vein, H: hepatocyte, LI: leukocyte infiltration, Dct: distal convoluted tubule, Pct: proximal convoluted tubule, Gl: glomerulus, ME: mesangial expansion, Br: bronchiole, Gr: granuloma, Ar: arteriole.

**Table 1:** Qualitative phytochemical screening of leaves aqueous extract of *O. celtidifolia*.

Classes of compound	*Opilia celtidifolia*
Phenols	−
Alkaloids	−
Flavonoids	−
Terpenoids	−
Tannins	+
Anthocyanins	−
Saponins	+
Proteins	−

+: presence; –: absence

**Table 2:** Quantity of some phytochemical compounds in the leaves aqueous extract of *O. celtidifolia*.

Compounds	Quantity in extract *of O. celtidifolia*	Units
Saponins	6.48 ± 1.27	Eqg Galac/100 g
Tannins	9.41 ± 3.63	Eqg Catech/100 g

**Table 3:** Effects of *O. celtidifolia* on body weight evolution.

Parameters	Normal control	Negative control (H_2_O, 5 ml/kg)	Positive control (silymarin, 100 mg/kg)	*O. celtidifolia* (100 mg/kg)	*O. celtidifolia* (200 mg/kg)	*O. celtidifolia* (400 mg/kg)
D1	97.83 ± 8.87	91.64 ± 8.21	112.00 ± 11.40	84.66 ± 5.70	92.33 ± 9.88	102.10 ± 14.00
D7	105.50 ± 10.05	101.65 ± 8.95	120.16 ± 12.54	91.16 ± 11.50	105.16 ± 9.39	110.33 ± 8.65
D14	116.33 ± 8.81	113.00 ± 5.75	126.16 ± 12.04	99.66 ± 4.65	112.33 ± 7.94	115.83 ± 8.56
D21	138.49 ± 10.39	127.66 ± 5.06	151.34 ± 14.35	129.00 ± 6.57	136.8 ± 6.84	134.8 ± 6.39

D1, D7, D14, and D21 correspond to the 1^st^, 7^th^, 14^th^, and 21^st^ days of treatment.

**Table 4:** Effects of *O. celtidifolia* on some parameters of liver function.

Parameters	Normal control	Negative control (H_2_O, 5 ml/kg)	Positive control (silymarin 100 mg/kg)	*O. celtidifolia* (100 mg/kg)	*O. celtidifolia* (200 mg/kg)	*O. celtidifolia* (400 mg/kg)
ALT (UI/l)	56.43 ± 3.42	76.83 ± 4.19^**###**^	39.4 ± 7.12	38.79 ± 5.42^∗∗∗^	26.42 ± 4.52^∗∗∗^	48.03 ± 7.22^∗∗^
AST (UI/l)	115.99 ± 22	192.38 ± 26.33^**#**^	125.5 ± 13.58	108.00 ± 8.19^∗∗^	80.5 ± 6.54^∗∗^^##^	120.54 ± 11.31
ALP (UI/l)	65.76 ± 7.31	88.38 ± 14.56^#^	82.51 ± 17.01	43.7 ± 3.02	42.10 ± 9.55	30.03 ± 5.28^∗∗∗^^##**#**^
Total bilirubin (mg/dl)	4.3 ± 0.032	5.15 ± 0.65	4.1 ± 0.77	2.85 ± 0.64^∗∗^^#^	1.53 ± 0.28^∗∗∗^^##^	2.75 ± 0.47^∗∗∗^^##^
Conjugated bilirubin (mg/dl)	0.145 ± 0.01	0.47 ± 0.15	0.49 ± 0.04	0.64 ± 0.16^∗∗^	0.76 ± 0.085	0.81 ± 0.50

The values represent mean ± SEM, ^∗∗^*p* < 0.01 significant statistical differences compared to the negative control. ##*p* < 0.01 significant statistical differences compared to the normal control.

**Table 5:** Effects of *O. celtidifolia* leaf aqueous extract on the lipid profile and total protein levels.

Parameters	Normal control	Negative control (H_2_O, 5 ml/kg)	Positive control (silymarin 100 mg/kg)	*O. celtidifolia* (100 mg/kg)	*O. celtidifolia* (200 mg/kg)	*O. celtidifolia* (400 mg/kg)
Total cholesterol (mg/dl)	88.33 ± 29.83	103.75 ± 4.50	880.01 ± 137.23	92.50 ± 11.04	74.26 ± 6.43	49.16 ± 4.35^∗∗ ##^
HDL cholesterol (mg/dl)	59.33 ± 3.16	50.25 ± 4.84	6774.23 ± 58.82	52.46 ± 11.13	49.32 ± 2.36	53.50 ± 1.58
LDL cholesterol (mg/dl)	30.30 ± 3.20	31.32 ± 3.32	1730.7284 ± 32.32	28.1 ± 2.9	26.32 ± 6.03	28.9 ± 3.22
Triglycerides (mg/dl)	71.81 ± 13.30	79.48 ± 7.34	69114.27 ± 153.28	43.45 ± 3.64	78.36 ± 4.12	42.12 ± 2.25^∗∗^
Total proteins (mg/dl)	87.17 ± 14.11	65.86 ± 4.83	1198.5713 ± 18.71	71.85 ± 6.18	74.17 ± 15.81	68.6 ± 11.63

The values represent mean ± SEM, ^∗∗^*p* < 0.01 significant statistical differences compared to the negative control. ##*p* < 0.01 significant statistical differences compared to the normal control.

## Data Availability

The data used to support the findings of this study are available from the corresponding author upon request.
